# How low can you go: evaluating electrode reduction methods for EEG-based speech imagery BCIs

**DOI:** 10.3389/fnrgo.2025.1578586

**Published:** 2025-07-02

**Authors:** Maurice Rekrut, Johannes Ihl, Tobias Jungbluth, Antonio Krüger

**Affiliations:** ^1^Cognitive Assistants Department, German Research Center for Artificial Intelligence, Saarbrücken, Germany; ^2^Saarland Informatics Campus, Saarbrücken, Germany

**Keywords:** BCI, speech imagery, imagined speech, EEG, electrode reduction

## Abstract

Speech imagery brain-computer interfaces (SI-BCIs) aim to decode imagined speech from brain activity and have been successfully established using non-invasive brain measures such as electroencephalography (EEG). However, current EEG-based SI-BCIs predominantly rely on high-resolution systems with 64 or more electrodes, making them cumbersome to set up and impractical for real-world use. In this study, we evaluated several electrode reduction algorithms in combination with various feature extraction and classification methods across three distinct EEG-based speech imagery datasets to identify the optimal number and position of electrodes for SI-BCIs. Our results showed that, across all datasets, the original 64 channels could be reduced by 50% without a significant performance loss in classification accuracy. Furthermore, the relevant areas were not limited to the left hemisphere, widely known to be responsible for speech production and comprehension, but were distributed across the cortex. However, we could not identify a consistent set of optimal electrode positions across datasets, indicating that electrode configurations are highly subject-specific and should be individually tailored. Nonetheless, our findings support the move away from extensive and costly high-resolution systems toward more compact, user-specific setups, facilitating the transition of SI-BCIs from laboratory settings to real-world applications.

## 1 Introduction

Speech Imagery Brain-Computer Interfaces (SI-BCIs) aim to decode imagined speech from brain activity and offer a variety of useful applications whenever spoken speech is not an option (Rekrut et al., 2022b). Although the feasibility of such BCIs has been proven in several studies, even for non-invasive brain measures such as electroencephalography (EEG) (Sereshkeh et al., 2017b; Nguyen et al., 2018), current EEG-based setups are complex and rely on high-resolution devices with 64 or more channels (Sereshkeh et al., 2017a; Lee et al., 2020). However, such high-resolution devices are expensive, cumbersome to wear, and inconvenient in everyday life. Existing approaches working with reduced sensor setups and a more compact and convenient design have so far failed to meet expectations regarding classification performance.

Kaongoen et al. tried to classify imagined speech with Ear-EEG, an acquisition technique that monitors EEG from around or inside the user's ears (Kaongoen et al., 2021, 2022). Their proposed system with four electrodes arranged behind each ear showed promising results in offline analysis with an average classification accuracy of 10 subjects of 38.2% when performing imagined speech of 4 words (Kaongoen et al., 2021). Although these results exceed the chance level, they are far from being applicable in real-world scenarios, and only a few participants were able to utilize the proposed system in online control (Kaongoen et al., 2022). As there is a significant gap between these extremely compact and extremely extensive setups in terms of classification performance, the question arises of how to strike a balance between a comfortable setup and adequate system performance to make SI-BCIs applicable for real-world applications. However, only a few studies have attempted to answer this question by systematically reducing the number of electrodes to identify the optimal subset. The majority of existing studies in the field focus on reducing the number of electrodes to a predefined subset based on assumptions about functional brain areas related to speech processing (Qureshi et al., 2017; Abdallah et al., 2018). Those studies compare the complete set of electrodes against 3 to 4 subsets of electrodes, primarily involving the Broca and Wernicke areas. Systematic approaches for electrode reduction in SI-BCIs, based on algorithms that explore the potential of different electrode subsets and their combinations, are rare.

In Torres-Garcìa et al. (2016), a systematic approach was presented that aimed to find the minimal subset of channels required for reliable imagined speech detection. The task involved imagining the five Spanish words: “up,” “down,” “left,” “right,” and “select.” EEG data were recorded with an Emotiv EPOC headset with 14 electrodes for 27 participants. The approach was based on a wrapper function with objectives of minimizing the error rate and the number of channels, where the error rate was confirmed using the results of a random forest classifier. The system selected 6 to 8 channels and achieved a maximum classification accuracy of 90% on the dataset of five classes. However, the use of the Emotiv EPOC for this purpose appears questionable due to several reasons. First, this device is a consumer-grade headset with limited signal quality and only 14 electrodes. Furthermore, the 14 electrodes are mainly placed in frontal areas and at the outer borders of the parietal, temporal, and occipital regions of the brain, excluding the central region. Although partially including speech-relevant areas, this setup appears too limited to get a clear picture of the relevant brain regions and electrode positions, especially considering that the vast majority of research on SI-BCIs uses 32 channels and more to record brain signals homogeneously spread over the scalp (Rekrut et al., 2020; Jahangiri et al., 2018; Jahangiri and Sepulveda, 2019; Lee et al., 2020; Sereshkeh et al., 2017a; Zhao and Rudzicz, 2015; Kim et al., 2014). Thus, besides the worse signal quality of a consumer-grade EEG headset compared to a clinical one, the low number of electrodes overall could be a limiting factor when conducting a systematic evaluation.

Although the current literature acknowledges the awareness of overly complex setups and the resulting lack of SI-BCIs in real-world applications, no study has yet systematically evaluated electrode reduction methods on a broad set of data. Existing studies in the field rely on the comparison of predefined subsets of electrodes (Qureshi et al., 2017; Abdallah et al., 2018), smaller or inadequate sensor positions (Kaongoen et al., 2021, 2022), and focus solely on one specific dataset for evaluation, neglecting the variability between different data recording setups.

Addressing these shortcomings, this study evaluates different electrode reduction algorithms on EEG data recorded during imagined speech from three datasets, acquired in three different study setups by three different research groups. Thus, we aim to provide a broader perspective and basis for evaluating the success of electrode reduction algorithms and their generalizability across different datasets to determine the most suitable subset of electrodes for EEG-based imagined speech detection.

Thereby, this study aims to provide three main contributions to the field of Speech Imagery BCIs:

- **Evaluation of electrode reduction methods in speech imagery BCIs**—investigate different methods for electrode reduction in combination with different feature extraction and classification methods to provide recommendations on the best performing approach.
- **Determining the optimal number of electrodes for imagined speech classification**—identify the ideal number of electrodes, analyze subject-specific variability and assess the trade-off between accuracy and electrode reduction.
- **Identifying key electrode positions for speech imagery BCIs**—examine the spatial distribution of the most relevant electrodes between subjects and discuss the implications for standard EEG configurations.

With these contributions, we aim to lay the foundation to improve practicality, reduce setup time, and lower costs for the currently cumbersome speech imagery BCI setups, thereby facilitating their application in real-world scenarios in the future.

## 2 Material and methods

Our evaluation included several electrode reduction methods from the literature, combined with various feature extraction and classification algorithms, which are presented in detail in this section. Due to the complexity of this setup, we decided to conduct a holistic evaluation on only one of the three datasets to determine the best configuration for imagined speech data. This method was then applied to the systematic evaluation of all three imagined speech datasets, to identify a specific subset of electrodes and electrode positions that are best suited for SI-BCIs, thereby demonstrating the generalizability of the method.

This section is structured according to the different steps of the processing pipeline, including preprocessing, feature extraction, classification, and finally, the electrode reduction procedure, as well as our evaluation criteria. We start by explaining the general concept and the datasets used for evaluation. Due to the similarity of methods between our exploratory approach to finding the best-suited algorithms and the subsequent systematic evaluation on three different datasets, we describe the different steps only once and highlight the differences between the two setups in the respective subsections.

### 2.1 Concept

**Figure 1:**
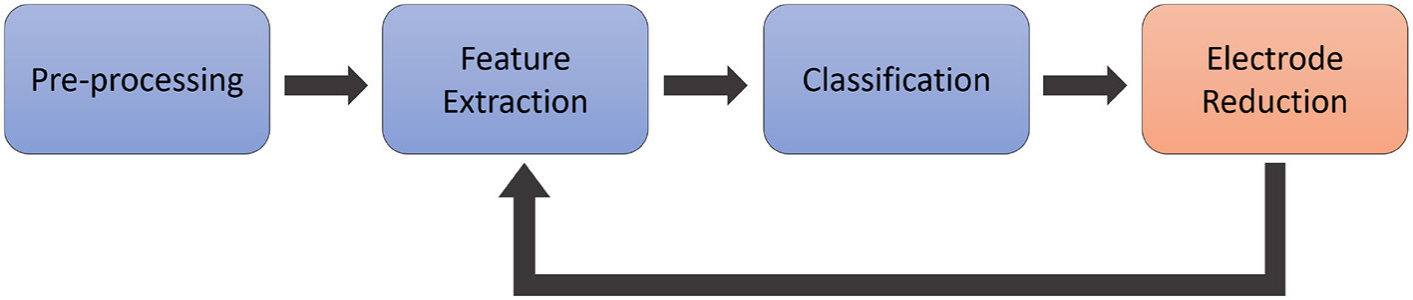
Conceptual illustration of the processing pipeline. The data is pre-processed in a first step, and afterwards, features are extracted and forwarded to a classifier. Finally, the electrode reduction method selects one electrode to be excluded, and the remaining data is forwarded again to the feature extraction.

Our literature research as described in the introduction led us to the conclusion to use wrapper methods due to their better performance as compared to filter and embedded methods (Maldonado and Weber, 2009; Kumari and Swarnkar, 2011), the ease of implementation, and the numerous existing implementations in previous research (Al Moubayed et al., 2010, 2012; Yang et al., 2012; Torres-Garcìa et al., 2016). These methods aim to assess specific channel subsets based on the accuracy achieved by the algorithm during learning. In our implementation, we systematically reduced the number of electrodes from the maximum to the minimum, as presented in Marx et al. (2019), based on the classification accuracy achieved with each subset. Hence, the data need to be processed with a standard pipeline for EEG-based imagined speech BCIs as illustrated in Figure 1. Our pipeline starts with preprocessing the data to remove artifacts and limit the time signal to a certain frequency spectrum relevant for analysis. In the next step, the features are extracted and forwarded to a classifier, which is trained to predict those features and is tested on a separate test set of data. After this classification, the electrode reduction algorithm selects an electrode to be excluded from the classification process. The data, reduced by the channel selected by the algorithm, is then forwarded again to the feature extraction, and this cycle continues until only one channel remains.

This procedure was implemented for all combinations of the different feature extraction, classification, and electrode reduction methods, and the intermediate results of the classification processes were stored for comparison.

### 2.2 Data

All three datasets consisted of EEG data recorded from at least 15 participants while performing imagined speech. In all studies, the EEG was recorded with 64 electrodes placed according to the 10-20 system and with the same hardware, the Brain Products Live-Amp.^1^ Although the datasets were recorded by different research groups, they differed only slightly in the recording procedure and the number of words used, as detailed in the following section.

#### 2.2.1 Dataset 1

Dataset 1 was taken from publicly available data of the 2020 international BCI competition.^2^ Participants repeated the 5 Korean words “Hello,” “Help me,” “Stop,” “Thank you” and “Yes,” overall, 70 times per word, while seated in a comfortable chair in front of a screen. Participants were advised not to engage in any other brain activity except for the given task, to remain still, and to avoid eye blinks during the imagined speech period. The stimulus presentation began with a 3-s initial rest period, followed by an audio cue of the target word. The cue presentation was followed by a randomly selected resting time of 0.8 to 1.2 s, during which a fixation cross was displayed. The subjects were then instructed to perform imagined speech of the given word four times before moving on to the next one. Stimulus presentation was randomized, and 70 repetitions per word were recorded, resulting in a dataset size of 350 trials overall per participant. The data was split into 300 trials for training and 50 trials for the test set. The trails for the test and train sets were predefined for the BCI competition and used accordingly for our evaluation. Information about the handedness of the participants was not provided in the dataset description.

#### 2.2.2 Dataset 2

Dataset 2 was acquired in the scope of the study presented in Rekrut et al. (2022a). It consists of the EEG data of 17 participants while they produced imagined speech of the 9 German words: “screw,” “case,” “circuit-board,” “floor,” “conveyor belt,” “workbench,” “push,” “hold,” and “lift.” Overall, 40 repetitions per word were recorded using a study setup similar to Dataset 1, with the only difference being that participants repeated each word only once. This procedure resulted in a dataset size of 360 trails per participant. For better comparability with the other two datasets, we randomly excluded two participants, resulting in a final analysis of 15 participants. All participants were right-handed. We applied a random train-test split with sklearn of 80/20 and used this initially created split throughout the overall procedure of the electrode reduction process. This approach was chosen to better compare with dataset 1 and the predefined test and train set.

#### 2.2.3 Dataset 3

Dataset 3 was taken from the study presented in Rekrut et al. (2022b). It consists of data from 15 right-handed participants silently repeating the five English words “up,” “left,” “right,” “push,” and “pick” to navigate a robot through a maze-like game on a computer screen. Using this setup, we collected 80 imagined repetitions per word, resulting in a dataset size of 400 trails per participant. More details on the data acquisition and the study itself can be found in Rekrut et al. (2022b), and the data is available on the Zenodo platform.^3^ We randomly split the data with sklearn into 80% train and 20% test set and used this initially created split throughout the overall procedure of the electrode reduction process for better comparability with dataset 1.

Although partially recorded under different circumstances, such as varying numbers of repetitions per word, the language and the words themselves, the overall important characteristics of the datasets are the same, namely, the number and position of electrodes used, the number of participants, the paradigm of imagined speech and even the recording hardware. Furthermore, the data were recorded from different participants for each study. This makes it the perfect setup for systematic evaluation of electrode reduction and important electrode positions in SI-BCIs.

### 2.3 Preprocessing

The data was bandpass-filtered between 0.5 and 60 Hz and notch-filtered again at 50 Hz to remove any overlying powerline noise. The parameters for the filtering methods were chosen according to our previous studies (Rekrut et al., 2021). After filtering, the data were divided into epochs of 2 s, starting from the onset of the fixation cross prior to silent repetition, to reduce the signal to the relevant sections containing the imagined speech.

### 2.4 Feature extraction

Feature extraction plays a crucial role in BCIs, and the best-performing methods vary significantly depending on the individual (Rekrut et al., 2021). Thus, we included a variety of feature extraction and classification methods and evaluated them in combination with electrode reduction algorithms to find a suitable setup for systematic electrode reduction in SI-BCI. We decided to include some of the most common feature extraction methods in BCI research in the comparison. We implemented common spatial patterns (CSP), discrete wavelet transform (DWT), and a feature vector used in one of our previous studies (Rekrut et al., 2021).

DWT was implemented using the PyWavelets library (Lee et al., 2019) for wavelet decomposition. As mother wavelet, we applied biorthogonal 2.2 (bior2.2) as suggested in Feng et al. (2019). The data were decomposed to the fourth level. Afterwards, a wavelet feature vector was created out of the data as presented in Torres-Garcìa et al. (2016). This method utilizes the maximum and minimum values of a given time series T, as well as its average and standard deviation, in conjunction with the relative wavelet energy of the signal.

The feature vector was implemented based on Rekrut et al. (2020) and Rekrut et al. (2021). The 13 features were chosen in the time and frequency domains, which included power spectral intensity and relative intensity ratio (alpha, beta, gamma, delta, and theta), Petrosian and Higuchi fractal dimensions, Hjorth parameters, spectral entropy, and skewness, fisher information, approximate entropy, detrended fluctuation analysis and hurst exponent. The features were extracted with the open-source Python module PyEEG (Bao et al., 2011).

The Common Spatial Pattern algorithm is frequently used in BCI applications (Khan et al., 2019; Nguyen et al., 2018) and applies a linear transformation to project the multi-channel EEG signal data to a lower-dimensional spatial subspace. In our implementation, we used the multiclass CSP algorithm as provided by the MNE Python library (Gramfort et al., 2013). As this algorithm provides a spatial filtering of the signal, we decided to use it, in addition to its base implementation, filtered by our feature vector and the wavelet decomposition, as described in the two other feature extraction methods above. Both methods were applied after performing the CSP on the signal and are referred to in the following as CSPfv for the feature vector and CSPwav for the wavelet decomposition combination. The standard implementation is referred to as CSP.

### 2.5 Classification

Similar to the feature extraction step, we used several classification methods in combination with the aforementioned feature extraction methods, which are commonly used in imagined speech research. We implemented a Random Forest (RF) and a Support Vector Machine (SVM) algorithm as presented in our previous studies (Rekrut et al., 2020, 2021). Furthermore, we integrated an Extreme Gradient Boosting (XGB) based on Chen and Guestrin (2016). It used the mean error as an evaluation metric and was instructed to stop if the mean error did not decrease for 10 rounds. The objective function was chosen to be SoftMax for multiple classes. We furthermore implemented a neural network (NN) as described in the study by Panachakel et al. (2020). This neural network features five hidden layers using ReLU and hyperbolic tangent activation functions, except for the final layer, which uses the sigmoid function. Dropout and batch normalization were applied between each hidden layer. The network was instructed to stop training once validation loss no longer decreased for five epochs. Categorical cross-entropy was used as a loss function and Adam as an optimizer.

### 2.6 Electrode reduction

Electrode reduction is a relevant topic not only in SI-BCIs but BCIs in general. It can be applied in different ways and is usually classified as a filter, wrapper or embedded method (Torres-Garcìa et al., 2016). The latter ones assess channel selection during the process of training and are specific to a given learning function (Guyon and Elisseeff, 2006). Filter methods do not use learning functions but rather measure the inherent features from the data and select the relevant channels based on those features (Lan et al., 2006). Wrapper methods aim to assess certain channel subsets based on the accuracy obtained by the algorithm during learning. Those wrapper methods will be the focus of our work, as they have been used successfully in the past in various studies (Al Moubayed et al., 2010, 2012; Yang et al., 2012; Torres-Garcìa et al., 2016) due to their better performance and ease of implementation (Maldonado and Weber, 2009; Kumari and Swarnkar, 2011).

For our comparison of electrode reduction methods in Speech Imagery BCIs, we identified three promising methods in related studies: Gray Wolf Optimization (GWO), Common Spatial Pattern-Rank (CSP-Rank), and Independent component analysis (ICA). These methods have already been used in a BCI context and have proven to work reasonably well with EEG data (Ghosh et al., 2019; Feng et al., 2019; Chaumon et al., 2015); however, they have not been applied to electrode reduction in imagined speech BCIs. In the following section, we will give a detailed overview of the three electrode reduction methods.

#### 2.6.1 Gray Wolf Optimization (GWO)

The Gray Wolf Optimization Algorithm is part of the family of evolutionary algorithms, which are based on the idea of the “survival of the fittest” (Ghosh et al., 2019). This principle was implemented in the algorithm by evaluating a fitness function, specifically the classification accuracy, using a dataset where a randomly selected electrode was excluded. This process was repeated for all electrodes consecutively, and the electrode, without which the classification achieved the highest accuracy, was finally rejected. This implementation is inspired by the hunting behavior of the Gray wolf, where the alpha animal guides the hunt to the prey, encircling it to reach the closest point to capture it (Emary et al., 2016). Although promising, this algorithm has only been utilized to date for feature selection in imagined speech (Ghosh et al., 2019) and not for electrode reduction.

#### 2.6.2 Common Spatial Pattern-Rank (CSP-rank)

The CSP-Rank electrode reduction is based on the Common Spatial Pattern algorithm. This method is usually applied for spatial filtering of EEG data in BCI applications but can also be modified to reduce the number of electrodes. CSP creates spatial filters for each class. CSP-Rank utilizes the filter matrix typically used for transformation to identify influential electrodes. For this purpose, the vector with the smallest and largest L1-norm in the matrix is extracted. These vectors consist of filter coefficients that assign weights to each electrode based on its respective influence on the class, implying that a feature with a larger absolute value is more important. Thus, electrodes corresponding to the largest remaining coefficient are added to the set of electrodes to be used in classification until a stopping condition is reached. A more detailed description of how CSP-Rank is computed can also be found in Feng et al.'s (2019) work.

We included the CSP-Rank algorithm in two different implementations: the multiclass CSP, as mentioned in the feature extraction section, and the One-Vs-All implementation. The multiclass variant was implemented using the MNE Python library (Gramfort et al., 2013). CSP was applied to the data from all classes after filtering and epoching. This resulted in the mixing matrix W, from which the two vectors with the most common information are selected, as it corresponds to EEG signals (Grosse-Wentrup and Buss, 2008). For all vectors v in W, each of which corresponds to one channel of the signal, the smallest absolute values are selected because they contain the least information related to classification. Thus, this electrode is rejected.

Another way of transforming the originally binary algorithm of CSP into a multiclass approach is the One-Vs-All method. In this implementation, the data is split up into pairs corresponding to their label. The dual-class CSP algorithm is then computed, resulting in sets of eigenvectors. As presented by Feng et al. (2019), from each set of eigenvectors, the largest and smallest are extracted, as they contain the most information on which electrodes are most relevant for their respective classes. Each vector in this set represents a spatial filter for EEG data, with values corresponding to a specific electrode. Thus, among all the vectors chosen, the one with the largest absolute value, *v*_*max*_, is selected, as it indicates that the corresponding electrode was most significant in distinguishing between the classes. The electrode corresponding to the position of *v*_*max*_ in the eigenvector is then added to the set of chosen electrodes. This process is repeated until a stopping condition is reached. The unselected electrodes are discarded. In this case, the stopping condition was reached when a certain threshold of electrodes was met. Because of the nature of the processing pipeline, as explained in the concept at the beginning of this section, the electrodes are always reduced by one. This caused the threshold to be always one less than the current number of electrodes.

The standard multiclass CSP for electrode reduction is referred to as CSP, and the One-Vs-All approach is referred to as oCSP.

#### 2.6.3 Independent component analysis (ICA)

The third electrode reduction method we included in our evaluation is typically applied to EEG data to remove artifacts: Independent Component Analysis (ICA). The ICA algorithm transforms a set of time-series data vectors X into their separate independent components S using a weight matrix W. Thus, the ICA problem can be formalized as *S* = *W*·*X*. This algorithm assumes that each vector x in X can be created by a linear mixture of n independent components. The weight matrix W can be derived from these vectors by searching the matrix that minimizes the mutual information of all vectors and thus allows for finding the linear mixture S (Hyvärinen and Oja, 2000). ICA is commonly used to remove artifacts, such as eyeblinks or heartbeats, from EEG data (Torres-Garcìa et al., 2016; Rekrut et al., 2020). This is done by identifying components belonging to artifacts in S. Afterwards, the respective vector in the weight matrix is set to be the 0-vector, thus removing the artifact when doing the inverse transform. However, these components can also be used to find information on the neural components of the signal as proposed by Chaumon et al. (2015). Chaumon et al. suggested that neural components are more likely to be found in the 13% largest components of the signal. Thus, similar to the CSP-Rank algorithm, one can try to rank the vectors within the ICA's mixing matrix W and reduce electrodes based on this ranking. This is done by identifying electrodes contributing strongly to components, preferring those that contribute strongly to the top 13%. We computed a score for each electrode based on its contribution to the overall signal, as follows.

For all component vectors *c*_*n*_ ∈ *C*, with *n* ∈ *N* and N being the total number of components, denote the ith position in the vector as $c_{n}^{i}$, then the score for each electrode is the result of the following equation:


(1) $$\begin{array}{l} Score_{Ele_{i}}=\overset{N}{\underset{n=0}{\sum }}ismax\left(c_{n}^{i}\right),\forall i\in \left[1,..,|c_{N}|\right] \end{array}$$

with the *ismax*-operation being defined as follows:


$$ismax\left(c_{n}^{i}\right)=\left\{ \begin{array}{c} 2,\;\text{if }c_{n}^{i}=\max\left(c_{n}\right)\wedge c_{n}\in L\;\\ 1,\;\text{if }c_{n}^{i}=max\left(c_{n}\right)\wedge c_{n}\notin L\;\\ 0,\;\text{otherwise}\; \end{array}\right.$$

with L being the set of the 13% largest components for a set of components C. Note that it does not matter which vector length is taken as reference in Equation 1, as all component vectors are equally long. We selected the electrodes with the largest score until a threshold was reached.

### 2.7 Evaluation criteria

Given that our implementation involved various combinations of feature extraction, classification, and electrode reduction methods for comparison, we needed to establish criteria for assessing the performance of these different methods. Our primary evaluation criterion was classification accuracy, calculated as the sum of correct classifications divided by the total number of classifications. Higher classification accuracy is then linked to better performance of the combined method. By comparing the classification accuracies of the different methods, we selected the top sets, which performed best on the data, consisting of the methods used for electrode reduction, feature extraction and classification, as well as the number of electrodes used in this set. This was supposed to give us insights into the best-suited number of electrodes for imagined speech classification, but also on the feature extraction and classification methods in those best-performing subsets. We further compared the achieved accuracies to the theoretical chance level calculated by dividing 100% by the number of classes in each dataset. As this method assumes an infinite number of predictions, we adjusted this threshold according to the size of the respective dataset as described in Combrisson and Jerbi (2015). We calculated the significance threshold based on this procedure and a *p*-value of 5% to be 24.29%, 14.44% and 24.00% for datasets 1, 2 and 3, respectively.

**Figure 2:**
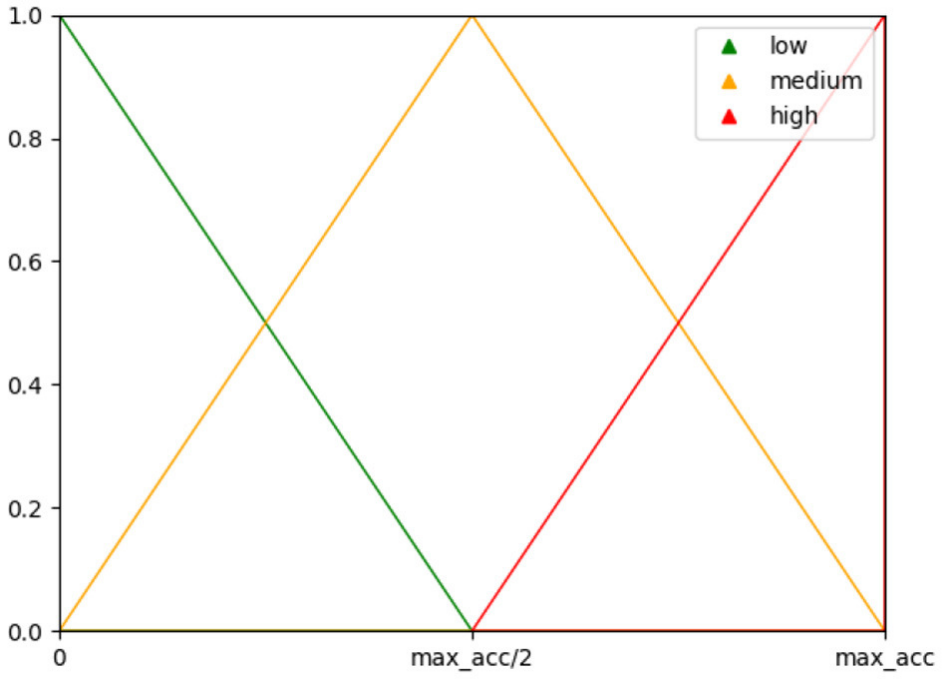
Membership functions used for the fuzzy inference system with the maximum classification accuracy as upper bound.

For our systematic evaluation of three datasets in the second part of the study, we further implemented a Fuzzy Inference System, as described in Torres et al. (2016). The purpose of this system was to prevent excluding sets with only slightly lower classification accuracy but a significantly lower number of electrodes. This system makes a decision based on a set of rules to provide a good balance between classification accuracy and the number of electrodes. The decision is based on the FIS membership function, which is computed from accuracy and the number of channels used and illustrated in Figure 2. In our implementation, we used the subject's maximum classification accuracy as an upper bound instead of the error rate used by Torres et al., since visual inspection of both methods yielded better results for the maximum accuracy, which led the FIS to not favor lower channel amounts for higher drops in accuracy.

Based on the top sets defined by our fuzzy inference system, we identified a possible optimal minimal number and subset of electrodes for SI-BCIs. Furthermore, we attempted to determine the most valuable electrode positions in the classification process by examining the frequency of occurrence of each electrode in the top sets per participant. We also applied k-means clustering to the top sets for all the data, and the data were split into different feature extraction methods to investigate valuable electrode positions in relation to a specific cluster of a lower or higher number of electrodes. Finally, we compared our results to subsets of electrodes chosen based on the functional areas of the cortex related to speech production, specifically all electrodes of the left hemisphere, the right hemisphere, and electrodes targeting Broca's and Wernicke's areas. The electrodes for Broca and Wernicke were selected according to Tsukahara et al. (2019) as “F7” and “P3.” Additionally, we included electrodes directly adjoining those two positions to cover a wider range of spatial information, resulting in F7, AF7, AF3, F5, F9, F3, FT9, FT7, FC5, CP5, CP3, CPz, P5, P1, P3, PO3, PO8, and POz.

## 3 Results

In the following section, we present the results of our evaluation of the performance of the different methods applied for feature extraction, classification, and electrode reduction, as well as the best-performing subsets of electrodes and their corresponding positions. Whenever we refer to the number of electrodes in this section in text and figure captions, it represents the number of electrodes removed from the set, not the remaining ones. We will repeat this fact several times throughout the text to avoid misunderstandings or misinterpretations.

### 3.1 Evaluation of electrode reduction methods

As mentioned in the previous section, our implementation involved combining various electrode reduction, feature extraction, and classification methods. The algorithms were trained on the data of each individual participant using a within-subject approach. We saved classification accuracies for all possible combinations of feature extraction and classification methods at each step of electrode reduction for evaluation. Our algorithm reduced the number of electrodes by one per iteration, employing four different electrode reduction algorithms. This resulted in a substantial collection of classification accuracies for all subjects, covering each subset of electrodes and each combination of feature extraction and classification methods. Given the vast number of different parameters, combinations of methods, and calculations, we decided to perform this evaluation only on dataset 1 and conclude on the best-performing setup for further assessment regarding a minimal subset of electrodes and their respective positions across all three datasets.

**Figure 3:**
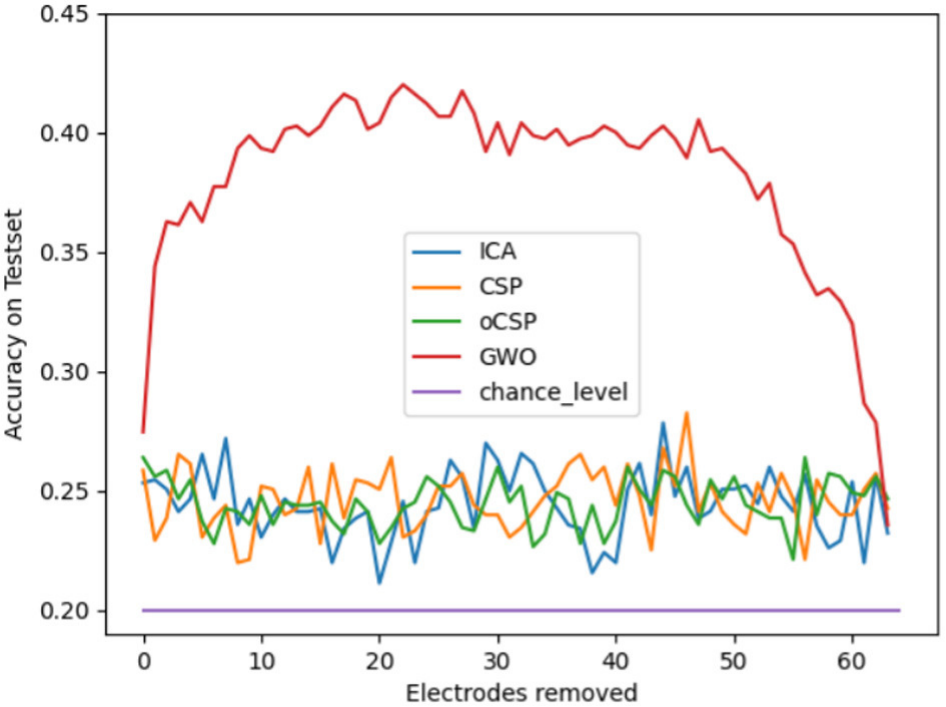
Mean classification accuracy on the test set across all subjects for all possible combinations of feature extraction and classification methods at each step of the different electrode reduction methods, presented as classification accuracy relative to the number of electrodes removed.

We initially calculated the mean classification accuracy on the test set for all participants in dataset 1, considering all possible combinations of feature extraction and classification methods at each stage of the various electrode reduction methods. The results of this calculation are shown in Figure 3 as the accuracy on the testset over the number of electrodes removed for the four different electrode reduction methods: ICA, CSP, oCSP, and GWO, as well as the chance level given at 20%. Notably, all methods scored consistently above chance level on average. Furthermore, the graph indicates that Gray Wolf Optimization massively outperformed the other methods by a large margin.

This impression was further strengthened by the analysis of individual data, which revealed that all best-performing setups, referred to as top sets, were generated by the GWO algorithm. An example of the results from the top sets is shown in Table 1 for all combinations of different feature extraction and classification methods for the best-performing subject 11. The left number in the brackets represents the classification accuracy achieved with the given configuration, and the number on the right represents the corresponding number of electrodes that achieved this accuracy.

**Table 1:** Example of individual top sets for all combinations of feature extraction and classification methods for GWO on the data of subject 11.

**Classifier**	**CSP**	**CSPfv**	**CSPwav**	**wav**	**featvec**	**Acc avg**	**Elec avg**
XGB	(0.45, 20)	(0.46, 24)	(0.56, 25)	(0.50, 53)	(0.34, 59)	0.464	36
NN	(0.38, 48)	(0.34, 43)	(0.36, 59)	(0.36, 49)	(0.36, 24)	0.36	45
RF	(0.44, 39)	(0.34, 58)	(0.56, 14)	(0.48, 36)	(0.28, 35)	0.42	36
SVM	(0.38, 15)	(0.40, 39)	(0.36, 62)	(0.42, 48)	(0.32, 63)	0.376	45
Acc avg	0.415	0.385	0.46	0.44	0.325	0.405	-
Elec avg	31	41	40	47	45	-	41

The left number in brackets represents the classification accuracy, and the right number corresponds to the number of electrodes for which this accuracy was achieved.

From the average classification accuracy concerning the given classifier on the right side of the table, it can be seen that XGB delivered the best classification accuracy.

Examining the results of all subjects, we found that the XGB clearly outperformed the other classification methods, with a 100% best performance on the GWO electrode reduction method and still above 50% at least for the remaining methods. A detailed view of the results showed that the XGB was chosen 45 times out of the 60 configurations resulting from the electrode reduction methods for 15 participants. This equals 75 % of all possible configurations. The NN classifier was chosen nine times, the RF classifier five times, and the SVM classifier only once.

The feature extraction methods did not show such a clear picture. In the GWO condition, DWT was considered the best-performing method, with 7 out of 15 occurrences in the top sets, followed by standard CSP with 4 occurrences and 3 for CSPwav. The feature vector was chosen only once, and the CSPfv method was not used at all. For the other electrode reduction algorithms, we found a relatively balanced distribution of methods, including CSPfv.

Due to the GWO massively outperforming the other electrode reduction methods and the outstanding performance of XGB with GWO, we decided to use this combination for further systematic analysis of all three datasets. The feature extraction did not provide a clear picture, and the best-performing methods varied individually between conditions and participants. To preserve these individual preferences, we selected the three best-performing feature extraction methods from the GWO configuration: CSP, DWT (wav), and the combination of the two, CSPwav. Since the feature vector was only responsible for a single top set in the GWO condition, and the combination of feature vector and CSP (CSPfv) did not correspond to any single one, we decided to exclude them from further investigation.

### 3.2 Optimal electrode count and subset selection

In the following section, we present the results of our systematic reduction of electrodes by applying GWO in combination with XGB and three different feature extraction methods, based on the results presented in Section 3.1 on three different imagined speech datasets described in Section 2.

**Figure 4:**
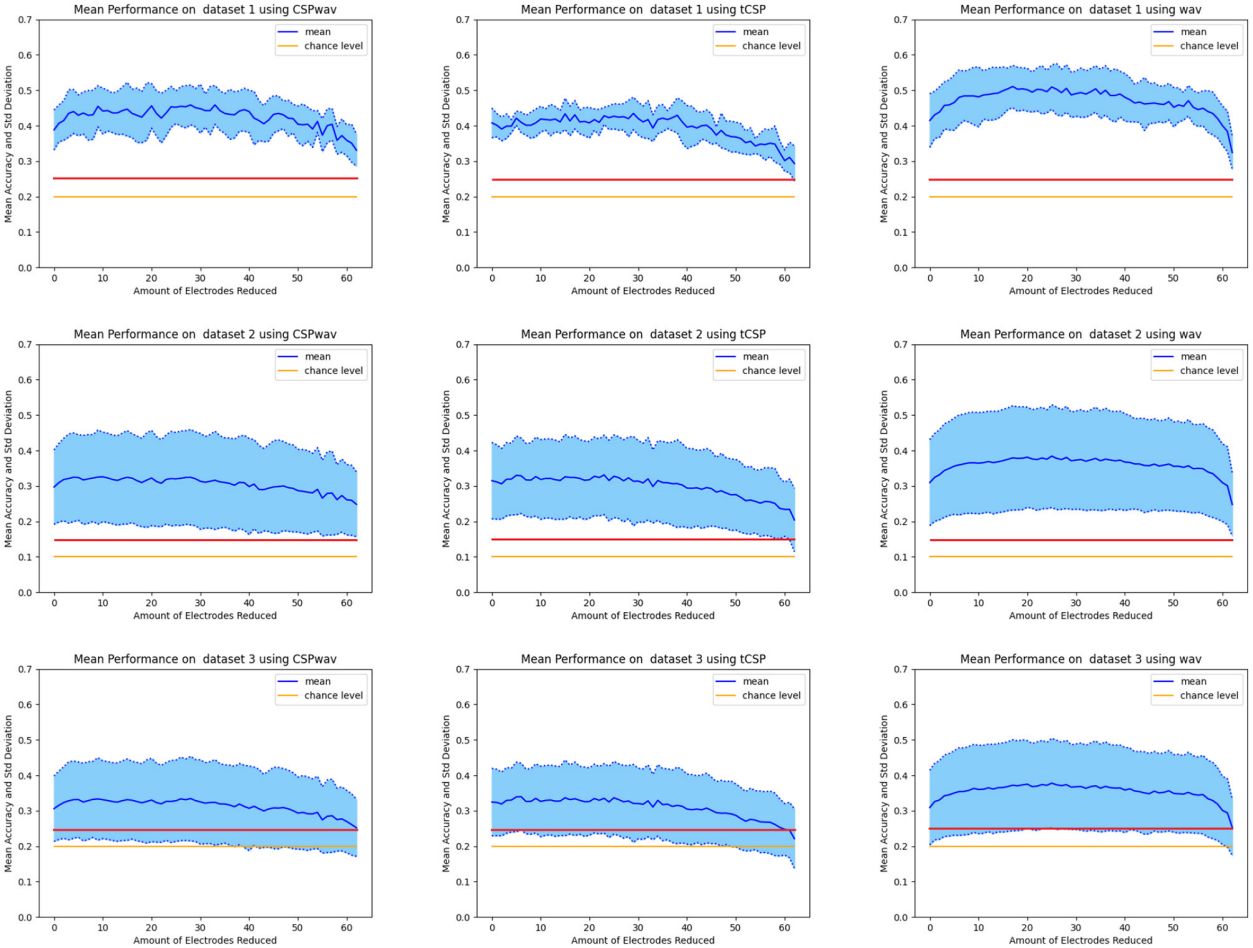
Mean classification accuracy of all participants over the number of electrodes removed. In the first row is dataset one, the second row is dataset two, and the third row is dataset three. The first column displays the results for CSPwav, the second column shows CSP, and the third column presents the DWT feature extraction method. The solid blue line represents the mean value, the yellow line represents the chance level, and the red line represents the significance threshold.

Figure 4 presents the average results for all participants, the three different datasets, and the three feature extraction methods, as well as classification accuracy over the number of electrodes removed. All diagrams clearly show the expected shape of an early increase in classification accuracy after reducing the first electrodes, followed by a decrease if too many electrodes are removed toward the end. This shape was most distinct for the discrete wavelet transform (wav) feature extraction method represented in the right column. It confirmed our results from the previous section and the success of GWO for electrode reduction on the two additional imagined speech datasets.

**Figure 5:**
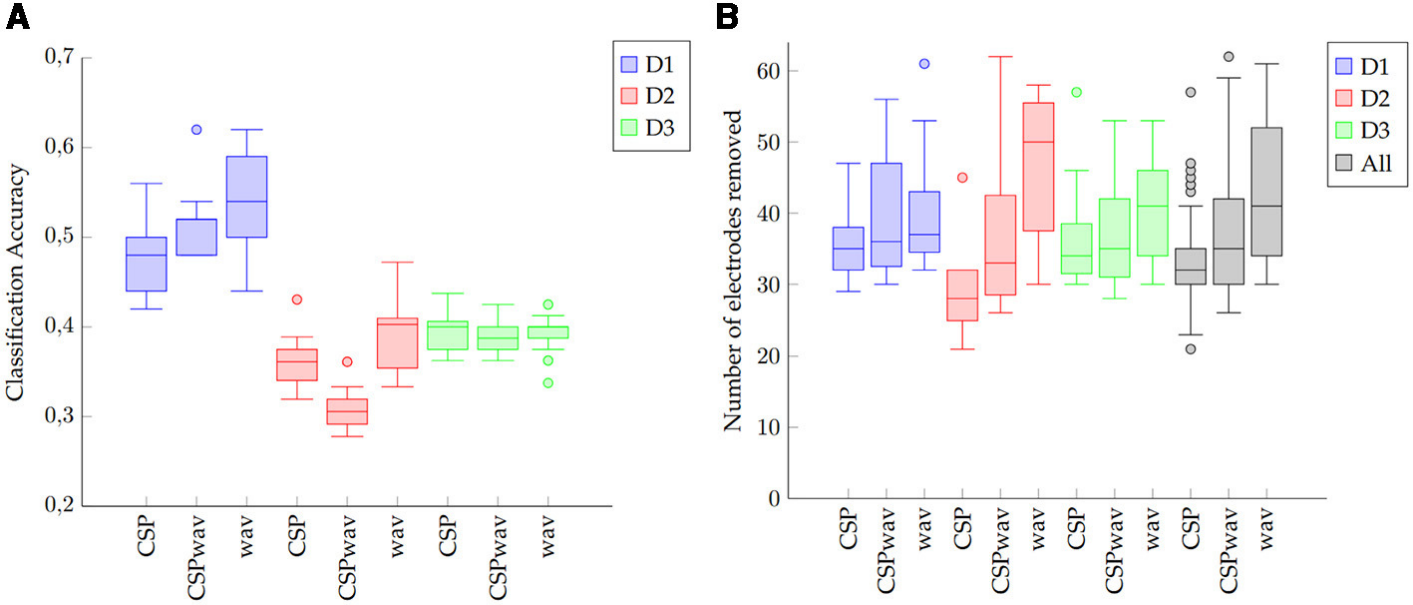
Boxplots of **(A)** classification accuracies and **(B)** the number of electrodes removed for each of the three datasets (D1, D2, and D3) and feature extraction methods. The black boxes on the right show the results for all datasets combined (All).

The detailed results for the individual top sets of each participant, determined by our fuzzy inference system for the three datasets and the three feature extraction methods, can be found in Tables 2–4. For dataset one, on average, 38 electrodes were removed, resulting in a classification accuracy of 51%, which is significantly above the chance level and the significance threshold of 24.29%. The average classification accuracies did not differ much between the three feature extraction methods; however, the boxplots, shown in Figure 5A, confirm the superior performance of the wavelet transform (wav) method, as indicated by the 54% average classification accuracy and the fact that in 12 out of 15 times, the highest classification accuracy was achieved with wav. The three remaining top sets resulted from the CSPwav feature extraction method. Examining the number of electrodes removed in the top sets, we observe that the average numbers do not differ significantly, but the values exhibit a rather high standard deviation, indicating that the numbers vary substantially between participants. Figure 5B illustrates this fact in the boxplots, showing a wide range of removed electrodes from 30 to 56 in the case of the CSPwav feature extraction method. Given this strong distribution and wide range of different numbers of electrodes removed, it is not possible to conclude on a single best subset or number of electrodes that would be suitable for all participants. However, the medians center ~36 electrodes removed.

**Table 2:** Top sets as determined by our fuzzy inference system for each participant and each feature extraction method in dataset one.

**Subject**	**CSP**	**CSPwav**	**wav**	**Acc avg**	**Elec avg**
1	(0.44, 47)	(0.54, 37)	(0.60, 37)	0.53 ± 0.08	40 ± 6
2	(0.42, 35)	(0.48, 48)	(0.50, 53)	0.47 ± 0.04	45 ± 9
3	(0.46, 32)	(0.48, 49)	(0.60, 33)	0.51 ± 0.08	38 ± 10
4	(0.56, 41)	(0.52, 33)	(0.58, 40)	0.55 ± 0.03	38 ± 4
5	(0.52, 40)	(0.52, 34)	(0.62, 36)	0.55 ± 0.05	37 ± 3
6	(0.48, 29)	(0.48, 33)	(0.54, 34)	0.50 ± 0.03	32 ± 3
7	(0.50, 31)	(0.52, 36)	(0.60, 34)	0.54 ± 0.05	34 ± 3
8	(0.42, 37)	(0.48, 30)	(0.44, 37)	0.45 ± 0.03	35 ± 4
9	(0.56, 38)	(0.62, 32)	(0.50, 61)	0.56 ± 0.06	47 ± 15
10	(0.44, 34)	(0.48, 38)	(0.58, 35)	0.50 ± 0.07	36 ± 2
11	(0.50, 35)	(0.52, 56)	(0.58, 44)	0.53 ± 0.04	45 ± 11
12	(0.48, 32)	(0.52, 46)	(0.44, 42)	0.48 ± 0.04	40 ± 7
13	(0.42, 35)	(0.48, 48)	(0.50, 53)	0.47 ± 0.04	45 ± 9
14	(0.50, 30)	(0.54, 30)	(0.54, 35)	0.53 ± 0.02	3 ± 3
15	(0.48, 38)	(0.50, 30)	(0.54, 32)	0.51 ± 0.03	33 ± 4
**Acc avg**	**0.48** **±** **0.05**	**0.51** **±** **0.04**	**0.54** **±** **0.06**	**0.51** **±** **0.05**	-
**Elec avg**	**36** **±** **5**	**39** **±** **8**	**40** **±** **9**	-	**38** **±** **7**

The number in brackets on the left represents the classification accuracy, and the number on the right represents the number of electrodes removed from the original set of 64, which led to this accuracy. Bold values highlight the average accuracy and average number of electrodes removed for each method.

**Table 3:** Top sets as determined by our fuzzy inference system for each participant and each feature extraction method in dataset two.

**Subject**	**CSP**	**CSPwav**	**wav**	**Acc avg**	**Elec avg**
1	(0.37, 32)	(0.30, 43)	(0.40, 55)	0.36 ± 0.05	43 ± 12
2	(0.38, 21)	(0.27, 47)	(0.36, 50)	0.34 ± 0.06	39 ± 16
3	(0.36, 32)	(0.36, 29)	(0.41, 30)	0.38 ± 0.03	30 ± 2
4	(0.43, 25)	(0.33, 33)	(0.33, 33)	0.37 ± 0.06	30 ± 5
5	(0.33, 23)	(0.29, 35)	(0.37, 32)	0.33 ± 0.04	30 ± 6
6	(0.38, 26)	(0.36, 62)	(0.45, 55)	0.40 ± 0.05	48 ± 19
7	(0.36, 25)	(0.31, 29)	(0.38, 58)	0.36 ± 0.03	37 ± 18
8	(0.33, 31)	(0.30, 27)	(0.40, 34)	0.35 ± 0.05	31 ± 4
9	(0.31, 45)	(0.29, 42)	(0.47, 56)	0.36 ± 0.10	48 ± 7
10	(0.36, 29)	(0.31, 28)	(0.34, 55)	0.34 ± 0.02	38 ± 15
11	(0.37, 23)	(0.31, 59)	(0.40, 41)	0.37 ± 0.04	41 ± 18
12	(0.34, 32)	(0.29, 36)	(0.33, 58)	0.32 ± 0.03	42 ± 14
13	(0.37, 28)	(0.29, 32)	(0.45, 44)	0.38 ± 0.08	35 ± 8
14	(0.33, 28)	(0.30, 26)	(0.40, 43)	0.35 ± 0.05	32 ± 9
15	(0.37, 32)	(0.27, 27)	(0.34, 57)	0.33 ± 0.05	39 ± 16
**Acc avg**	**0.36** **±** **0.04**	**0.31** **±** **0.03**	**0.39** **±** **0.04**	**0.36** **±** **0.04**	-
**Elec avg**	**29** **±** **6**	**37** **±** **11**	**47** **±** **11**	-	**38** **±** **12**

The number in brackets on the left represents the classification accuracy, and the number on the right represents the number of electrodes removed from the original set of 64, which led to this accuracy. Bold values highlight the average accuracy and average number of electrodes removed for each method.

**Table 4:** Top sets identified by our fuzzy inference system for each participant and each feature extraction method in dataset three.

**Subject**	**CSP**	**CSPwav**	**wav**	**Acc avg**	**Elec avg**
1	(0.41, 34)	(0.42, 41)	(0.38, 33)	0.41 ± 0.02	36 ± 4
2	(0.36, 33)	(0.40, 31)	(0.38, 35)	0.38 ± 0.02	33 ± 2
3	(0.40, 57)	(0.42, 31)	(0.38, 48)	0.40 ± 0.02	45 ± 13
4	(0.37, 32)	(0.36, 35)	(0.36, 41)	0.37 ± 0.01	36 ± 5
5	(0.41, 31)	(0.37, 28)	(0.40, 33)	0.40 ± 0.02	31 ± 3
6	(0.38, 33)	(0.38, 42)	(0.40, 43)	0.39 ± 0.01	39 ± 6
7	(0.36, 34)	(0.40, 42)	(0.38, 41)	0.38 ± 0.02	39 ± 4
8	(0.38, 34)	(0.37, 29)	(0.33, 30)	0.37 ± 0.03	31 ± 3
9	(0.37, 43)	(0.37, 53)	(0.40, 30)	0.38 ± 0.01	42 ± 12
10	(0.40, 31)	(0.36, 35)	(0.40, 44)	0.39 ± 0.02	37 ± 7
11	(0.40, 44)	(0.38, 48)	(0.37, 53)	0.39 ± 0.01	48 ± 5
12	(0.43, 34)	(0.40, 42)	(0.41, 52)	0.42 ± 0.02	43 ± 9
13	(0.41, 46)	(0.36, 30)	(0.42, 36)	0.40 ± 0.03	37 ± 8
14	(0.40, 30)	(0.41, 33)	(0.40, 52)	0.40 ± 0.01	38 ± 12
15	(0.37, 30)	(0.37, 33)	(0.40, 35)	0.38 ± 0.01	33 ± 3
**Acc avg**	**0.39** **±** **0.02**	**0.38** **±** **0.02**	**0.39** **±** **0.02**	**0.39** **±** **0.02**	-
**Elec avg**	**36** **±** **8**	**37** **±** **7**	**40** **±** **8**	-	**38** **±** **8**

The number in brackets on the left represents the classification accuracy. In contrast, the number on the right represents the number of electrodes removed from the original set of 64, leading to this accuracy. Bold values highlight the average accuracy and average number of electrodes removed for each method.

Dataset two shows a similar behavior to dataset one. Again, we see an average of 38 electrodes removed from the initial set of 64, but with a higher standard deviation compared to dataset one. Classification accuracies are stable and do not differ too much, with an average classification accuracy of 36%. Looking at the boxplots in Figure 5A, we can see that although the average values let us conclude on a rather dense distribution, the wavelet feature extraction (wav) outperformed the other two methods. However, given the significance threshold of 14.44% those results are all significantly above the chance level for the nine words to be distinguished. The highest classification accuracies in this case were produced 10 times by the WAV feature extraction method and in the remaining five times by the CSP method. The boxplots for the number of removed electrodes in Figure 5B show an even clearer picture of the broad distribution of different numbers than in dataset one. There is no clear evidence on a certain number to remove to receive the best classification results. In this case, even the median value differs significantly between the different feature extraction methods, and we cannot determine a single best subset or optimal number of electrodes. Distinguishing between feature extraction methods, we can see a preference for the Wav method to remove more electrodes, while the CSP-based setups appear to remove less.

In dataset three, we can again observe similar figures for the average values in Table 4. Average classification accuracies cluster closely at ~39% and exhibit a low standard deviation. In the boxplot in Figure 5A, we can this time confirm the average results from the table, as the classification accuracies do not differ significantly, and apart from a few outliers for the wavelet feature extraction method (wav), the accuracies appear to concentrate around the average value of 39%. The highest classification accuracies were achieved six times by the CSP method, five times by the CSPwav, and four times by the wav feature extraction method.

Examining the number of electrodes removed, we once again observe an average of 38. However, the boxplot also reveals a wide distribution of the numbers among the various subjects, with no clear evidence of a specific number that might be suitable for all participants. Similar to Dataset 2, we can ascertain a preference for wave feature extraction using fewer electrodes compared to CSP-based methods.

This impression is confirmed by the black boxplots on the right in Figure 5B, which provide an overview of the distribution of the number of reduced electrodes per feature extraction method for the data from all three datasets. We can observe a tendency for the wavelet transform (wav) to reduce the number of electrodes more than the other two feature extraction methods. Although there appears to be a wide range, with some outliers, the interquartile range of the different methods indicates that the CSP and CSPwav feature extraction methods tend to use more electrodes than the wavelet transforms. Furthermore, we can observe that the top sets of the wavelet transform for all the participants in all datasets lie above 30 removed electrodes, which means that for this feature extraction method, we could have achieved the top set configurations with only 34 of the initial 64 electrodes. This represents a significant saving of electrodes and therefore effort in terms of setup times for imagined speech experiments. Although the CSP-based feature extraction methods appear to prefer more electrodes to achieve their top sets, the first quartile for both implementations ends up at 30 electrodes removed. This means that for 75% of the participants, we would have achieved the top results by reducing 30 electrodes, even for CSP-based feature extraction methods. Taking the numbers for all three methods together, we can include 83% of the top sets overall and therefore conclude that in our study, with three different datasets, we could have achieved the same results with roughly half the number of electrodes.

### 3.3 Identifying key electrode positions

To determine the relevant electrode positions in the defined top sets, we counted the occurrences of each individual electrode within those sets. The resulting values were calculated based on the number of times the algorithm selected the specific electrode in the classification process within the top set for all participants. We decided not only to examine all the top sets combined but also to cluster the sets according to electrode occurrence and gain an impression of possible hotspots in sets with a higher or lower overall number of electrodes. We therefore applied k-means clustering on the top sets of all participants and within the top sets of the three different feature extraction methods. The number of clusters for each set was determined by plotting the sum of squared errors (SSE) over the first 10 clusters and identifying the elbow point, which was found to be at three, resulting in three clusters for each set of electrodes.

**Figure 6:**
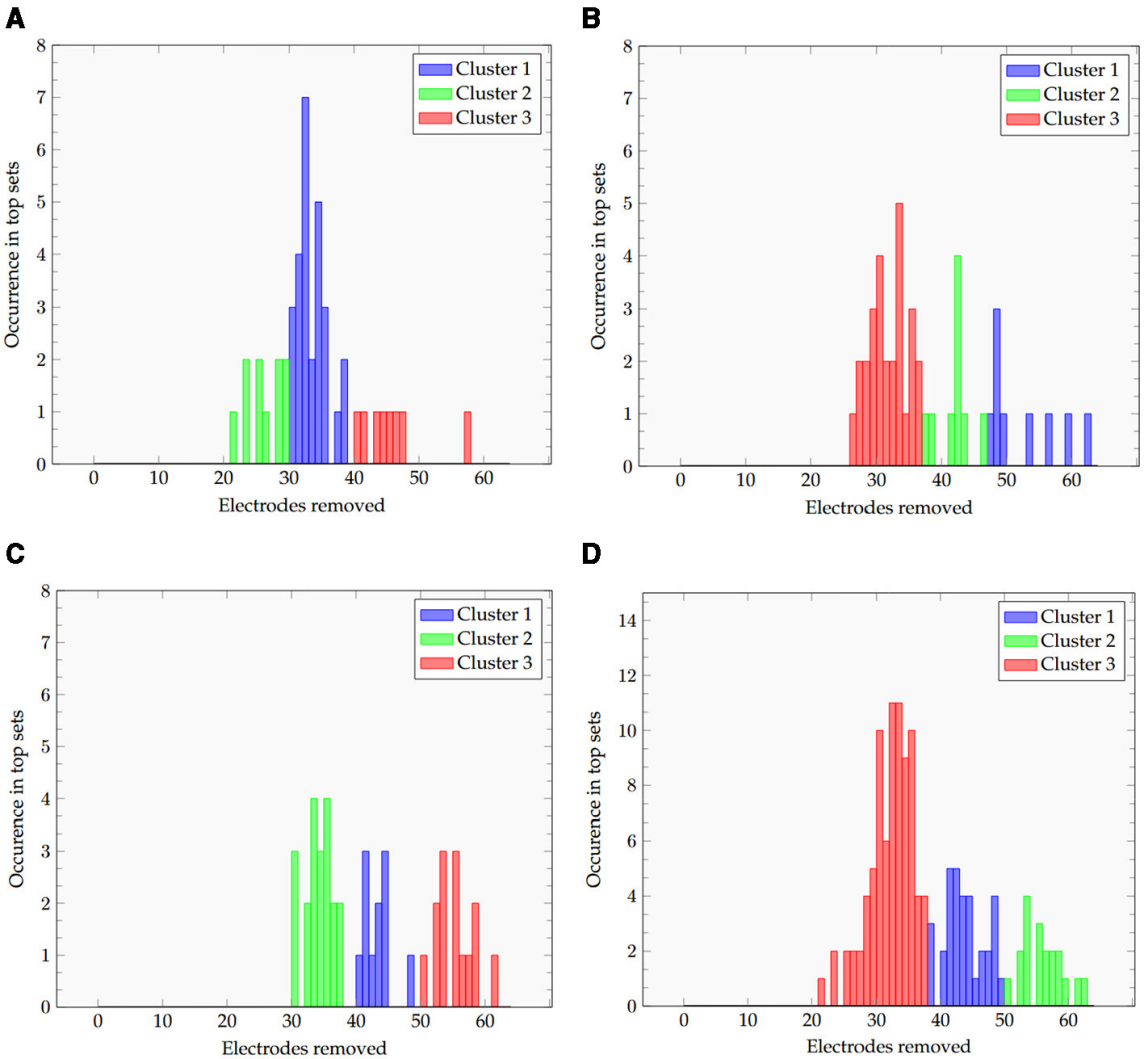
Histogram of the removed electrodes and the resulting 3 clusters for all datasets and the **(A)** CSP, **(B)**, CSPwav, **(C)** wav and **(D)** all feature extraction methods.

The results of the clustering are shown in Figures 6A–D. For each of the plots, we can clearly see clusters for low, medium, and high numbers of electrodes removed. Methods, including the wavelet transform (wav and CSPwav), appear to create clusters ~30, 40, and 50 electrodes, while the pure CSP appears to be separable into clusters at 25, 35, and 45, once more indicating the need for more electrodes in the classification process with those feature extraction methods.

**Figure 7:**
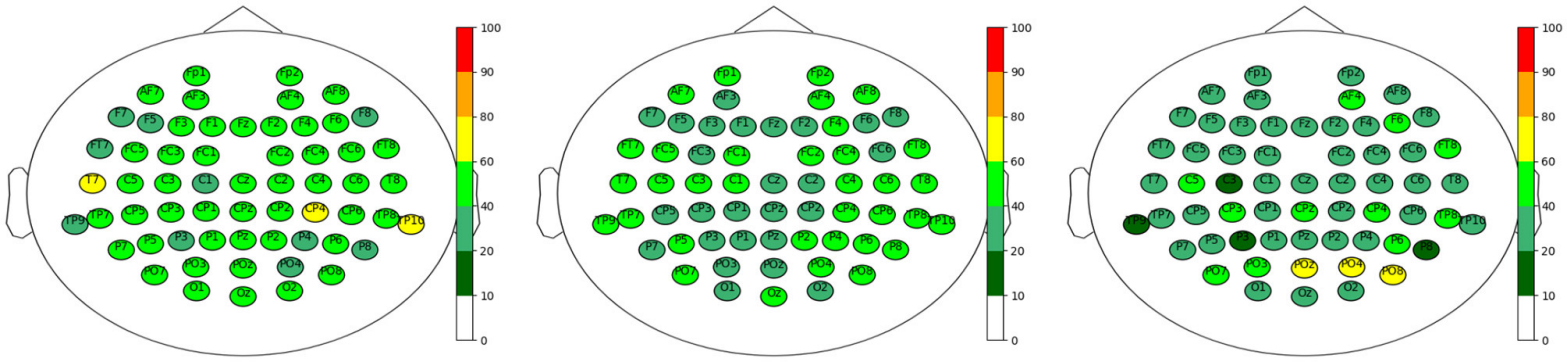
Electrode positions for the top sets of all three datasets and the three different feature extraction methods in percent. **Left**: CSP, **Middle**: CSPwav and **Right**: wav.

A clear conclusion on the relevant positions of electrodes could not, however, be drawn. For example, we have included the results for the electrode positions in the top sets of all datasets, separated by the feature extraction methods, in Figure 7. Each electrode position is visualized at its position on the head, colored according to the percentage of occurrence in the top sets as given in the legend on the right. We can observe a rather homogeneous distribution for all three feature extraction methods, which does not allow a clear conclusion on a certain brain region being dominantly involved in the classification process.

**Figure 8:**
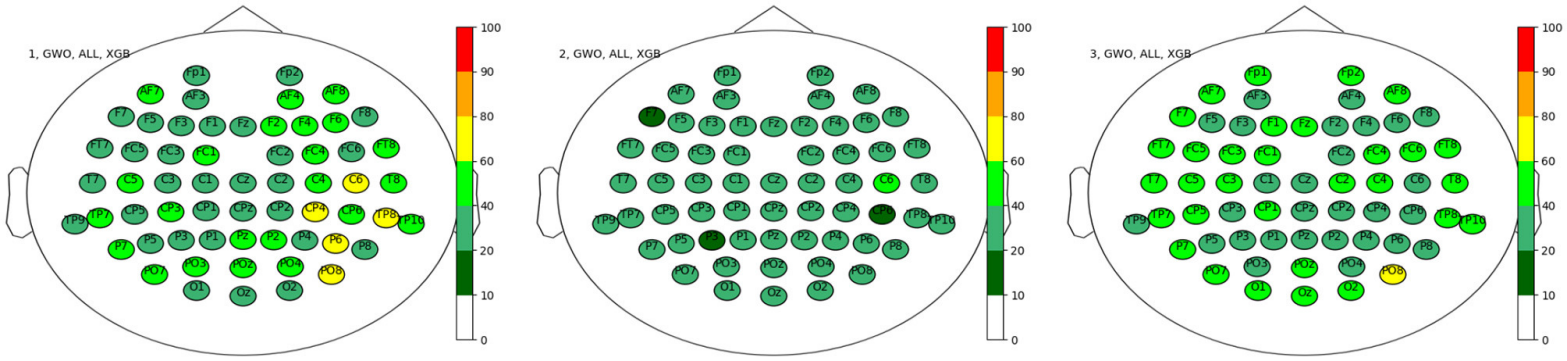
Electrode positions for the top sets separated by the three datasets in percent. **Left**: Dataset 1, **Middle**: Dataset 2 and **Right**: Dataset 3.

One could conclude that the occipital region plays a more dominant role in the case of the discrete wavelet transform; however, none of them passed the 80% threshold, and the remaining electrodes are spread rather homogeneously. This homogeneous distribution is further represented in the results for the different datasets shown in Figure 8, although one could conclude on a more dominant role of the right hemisphere of the brain for dataset 1, with increased activity around the parietal region.

Furthermore, these findings align with our classification results based on specific subsets representing functional areas of the cortex related to speech production. Table 5 shows the average results for these subsets, namely, all electrodes of the left hemisphere (Left) and electrodes targeting Broca and Wernicke areas (B & W), in comparison to the average results of our Gray Wolf Optimization algorithm. For the sake of completeness, we furthermore included all electrodes of the right hemisphere as a subset for comparison. The numbers for the specific subsets represent the overall best value from the three different feature extraction methods used. The results for the GWO on the left are presented in detail for all three feature extraction methods, facilitating a comparison of the values with the highest and lowest performance of the implementation. Within this table, the results clearly show that the GWO implementation outperformed the predefined subsets of electrodes from the left and right hemispheres as well as the Broca-Wernicke area with a difference in average classification accuracies of 20% for dataset one, 18% for dataset two, and 11% for dataset three. This was not only the case for the average values but also for all participants and individual accuracies, as shown in Table 6, which clearly supports the results that these electrode positions in the subsets are widely distributed across the cortex. This hypothesis is further supported by the fact that classification on the electrodes of the left and right hemispheres alone yielded equal average classification results for all datasets, indicating that both hemispheres contributed equally to the classification process on average. Thus, we conclude once more that the position of the electrodes is highly subject-specific and cannot be generalized to apply in a cross-subject condition.

**Table 5:** Average classification accuracies and remaining electrodes for the top sets of the GWO, categorized by feature extraction method (CSP, CSPwav, wav), electrodes of Broca and Wernicke (B & W) and the left and right hemisphere.

**D1**	**CSP**	**CSPwav**	**wav**	**B & W**	**Left**	**Right**
Acc avg	0.48	0.51	0.54	0.31	0.34	0.34
Elec avg	28	25	24	18	35	35
**D2**	**CSP**	**CSPwav**	**wav**	**B & W**	**Left**	**Right**
Acc avg	0.36	0.31	0.39	0.19	0.21	0.21
Elec avg	35	27	17	18	35	35
**D3**	**CSP**	**CSPwav**	**wav**	**B & W**	**Left**	**Right**
Acc avg	0.39	0.38	0.39	0.27	0.28	0.28
Elec avg	28	27	24	18	35	35

**Table 6:** Best top sets for each individual compared to the Broca and Wernicke as well as left and right hemisphere configuration of electrodes.

		**Dataset 1**				**Dataset 2**				**Dataset 3**			
**Nr**	**Param**	**GWO**	**B & W**	**Left**	**Right**	**GWO**	**B & W**	**Left**	**Right**	**GWO**	**B & W**	**Right**	**Left**
1	Acc	0.60	0.36	0.44	0.38	0.40	0.24	0.22	0.17	0.42	0.26	0.28	0.25
	Elec	37	18	35	35	55	18	35	35	41	18	35	35
	Feat	wav	CSPw	wav	wav	wav	CSPw	wav	CSP	CSPw	wav	wav	CSPw
2	Acc	0.50	0.26	0.32	0.28	0.38	0.22	0.18	0.22	0.40	0.29	0.23	0.36
	Elec	53	18	35	35	21	18	35	35	31	18	35	35
	Feat	wav	wav	wav	wav	CSP	wav	CSP	wav	CSPw	CSP	wav	wav
3	Acc	0.60	0.3	0.32	0.40	0.41	0.18	0.21	0.19	0.42	0.24	0.29	0.30
	Elec	33	18	35	35	30	18	35	35	31	18	35	35
	Feat	wav	wav	wav	wav	wav	CSPw	wav	CSP	CSPw	wav	CSP	CSPw
4	Acc	0.58	0.32	0.38	0.42	0.43	0.24	0.22	0.17	0.37	0.31	0.25	0.30
	Elec	40	18	35	35	25	18	35	35	32	18	35	35
	Feat	wav	CSP	wav	wav	CSP	CSP	wav	CSP	CSP	CSP	CSP	CSPw
5	Acc	0.62	0.38	0.28	0.30	0.37	0.15	0.22	0.19	0.41	0.25	0.29	0.26
	Elec	36	18	35	35	32	18	35	35	31	18	35	35
	Feat	wav	CSPw	wav	CSPw	wav	CSPw	CSPw	CSP	CSP	CSPw	CSPw	CSPw
6	Acc	0.54	0.3	0.32	0.34	0.45	0.18	0.24	0.24	0.40	0.2	0.29	0.24
	Elec	34	18	35	35	55	18	35	35	43	18	35	35
	Feat	wav	wav	wav	wav	wav	CSP	CSP	wav	wav	CSP	CSP	wav
7	Acc	0.60	0.34	0.34	0.40	0.38	0.18	0.17	0.29	0.40	0.31	0.28	0.26
	Elec	34	18	35	35	58	18	35	35	42	18	35	35
	Feat	wav	CSPw	CSPw	wav	wav	wav	wav	wav	CSPw	CSPw	CSP	wav
8	Acc	0.48	0.36	0.34	0.24	0.40	0.19	0.24	0.21	0.38	0.31	0.24	0.29
	Elec	30	18	35	35	34	18	35	35	34	18	35	35
	Feat	CSPw	CSPw	wav	CSP	wav	wav	wav	CSP	CSP	CSP	CSPw	CSP
9	Acc	0.62	0.36	0.4	0.34	0.47	0.21	0.17	0.18	0.40	0.24	0.28	0.25
	Elec	32	18	35	35	56	18	35	35	30	18	35	35
	Feat	CSPw	wav	CSPw	CSPw	wav	CSP	wav	wav	wav	CSP	wav	CSP
10	Acc	0.58	0.28	0.42	0.42	0.36	0.18	0.19	0.25	0.40	0.25	0.25	0.26
	Elec	35	18	35	35	29	18	35	35	31	18	35	35
	Feat	wav	CSP	wav	wav	CSP	CSP	wav	wav	CSP	wav	wav	CSPw
11	Acc	0.58	0.28	0.3	0.38	0.40	0.19	0.19	0.17	0.40	0.28	0.29	0.23
	Elec	44	18	35	35	41	18	35	35	44	18	35	35
	Feat	wav	CSPw	wav	wav	wav	CSP	CSP	CSP	CSP	CSP	CSP	CSPw
12	Acc	0.52	0.24	0.36	0.30	0.34	0.21	0.22	0.21	0.43	0.32	0.29	0.26
	Elec	46	18	35	35	32	18	35	35	34	18	35	35
	Feat	CSPw	wav	CSP	CSPw	CSP	CSP	wav	wav	CSP	CSPw	CSPw	CSPw
13	Acc	0.50	0.26	0.32	0.28	0.45	0.18	0.19	0.26	0.42	0.31	0.29	0.31
	Elec	53	18	35	35	44	18	35	35	36	18	35	35
	Feat	wav	wav	wav	wav	wav	wav	wav	wav	wav	wav	wav	CSP
14	Acc	0.54	0.3	0.34	0.36	0.40	0.17	0.21	0.22	0.41	0.23	0.3	0.34
	Elec	30	18	35	35	43	18	35	35	33	18	35	35
	Feat	CSPw	CSP	CSP	CSPw	wav	CSP	CSPw	CSP	CSPw	CSP	wav	CSP
15	Acc	0.54	0.36	0.28	0.30	0.37	0.19	0.25	0.18	0.40	0.26	0.28	0.25
	Elec	32	18	35	35	32	18	35	35	35	18	35	35
	Feat	wav	wav	wav	wav	CSP	wav	wav	wav	wav	wav	wav	wav
Avg	Acc	0.56	0.31	0.34	0.34	0.40	0.19	0.21	0.21	0.40	0.27	0.28	0.28
	Elec	38	18	35	35	wav	18	35	35	35	18	35	35
	Feat	wav	wav	wav	wav	wav	CSP	CSP	CSP	wav	CSP	wav	CSPw

## 4 Discussion

### 4.1 Evaluation of electrode reduction methods

In our attempt to find the best-performing method for electrode reduction in Speech Imagery BCIs, we can clearly state that the GWO algorithm outperformed the other implemented methods. A certain dominance of this algorithm was expected, as it evaluates the contribution of each electrode to the overall classification by calculating the accuracy for every subset after removing one electrode. Thus, the method is based on brute force and examines the data set down to the smallest detail, which makes it computationally intensive, but on the other hand, very precise. Furthermore, it showed the expected shape of the curve in Figure 3 for classification accuracy as a function of the number of electrodes removed. One would expect an initial rise in accuracy, as not all electrodes contribute equally well to the classification process, and some may be corrupted by noise or contain information irrelevant to the imagined speech production. After a certain number of electrodes were removed, the data no longer contained sufficient information, and the classification accuracy dropped again, as observed in Figure 3 at ~45 electrodes removed. This effect is surely influenced by various factors, such as artifact cleaning and filtering methods. Given a more sophisticated cleaning of the data, including ICA for artifact removal and more complex spatial filtering, this effect might vanish. In the case of our analysis, no ICA was applied because it was already used for electrode reduction in the later step of the analysis, which might have contributed to this characteristic shape, especially for the averaged results of all feature extraction methods and classifiers in Figure 3. However, compared to GWO, no other algorithms showed this behavior and remained in the lower classification accuracy range, oscillating around a value of ~25% classification accuracy without a clear indication of the peak or best setup. One could conclude on a peak for CSP and ICA at ~27% and 45 electrodes removed. In comparison to the clear difference from the GWO, this can, however, be neglected. Since this expected behavior was not present in the ICA, CSP and oCSP electrode reduction methods, and the significantly worse classification accuracy compared to the GWO algorithm, our clear recommendation for electrode reduction in imagined speech BCIs and the given setup is the GWO method.

Regarding the classifier, XGB clearly outperformed the other methods, especially when combined with the GWO electrode reduction method. In this case, all of the best-performing configurations used this classifier. As mentioned previously, this method calculates the accuracy for each subset and therefore provides a perfect basis for evaluating the classifier. But also for the other electrode reduction method, we can see a clear preference toward the XGB with its overall 75% appearance in the top sets without any real competitor among the other three classifiers. In this circumstance and the fact that XGB scored all the top sets in the GWO condition, our recommendation is with XGB for electrode reduction in speech imagery BCIs.

The results on feature extraction methods appear to support our findings from a previous study (Rekrut et al., 2020, 2021) as they are highly individual. For the GWO, one could claim a preference toward the wavelet decomposition, closely followed by the CSP algorithm and the CSPwav. Considering the other electrode reduction methods, however, there is no clear evidence for a preference for a specific feature extraction method. The occurrence in the top sets is rather balanced, and it appears to depend highly on the individual. To preserve these individual preferences, we recommend evaluating different methods on the individual's data. However, we would limit the selection in our study to CSP, DWT (wav), and the combination of the two, CSPwav.

### 4.2 Optimal electrode count and subset selection

The results presented in the previous section showed that it was not possible to determine one specific subset or number of electrodes that can be applied as a standard setup for EEG-based imagined speech BCIs. Although, on average, 38 electrodes were removed in the three datasets, we observe a strong variation among subjects with respect to the number of electrodes. Upon examining the three feature extraction methods, it can be concluded that feature extraction using the Discrete Wavelet Transform requires fewer electrodes compared to the CSP-based methods, which, on average, appear to include more electrodes in the top sets for classification. However, a concrete number could not be determined, as the results vary and are highly subject-specific. What can be concluded concerning the number of electrodes is that the 64-channel setup as used in all three studies appear to be oversized. Figure 5A illustrates that we could clearly reduce the number of electrodes for each participant by 20 without excluding any of the top sets determined by our fuzzy inference system. Furthermore, this was the case in all three different imagined speech datasets evaluated in our study. Increasing this number to 30 would still include all top sets of the wavelet feature extraction method and 75% of the two CSP-based methods, which shows that EEG-based imagined speech BCIs appear to work perfectly fine with smaller electrode setups. Our recommendation in this case is to limit further studies on imagined speech to setups with 32 electrodes, as they appear to have worked reasonably well for the three investigated datasets in our study. Starting with a higher number of electrodes and systematically reducing it to the best-performing subset of the individual appears necessary, as evidenced by the high variation in the remaining results above 30 electrodes removed. In the individual case, the number could be reduced even further.

### 4.3 Identifying key electrode positions

Similar to the results regarding the electrode count, the findings on specific electrode positions did not yield a clear picture either. Although the different numbers of electrodes for the individual top sets could be clearly divided into separate clusters for low, medium, and high numbers of electrodes, these clusters did not reveal specific positions on the cortex that could be seen as significantly contributing to the classification process. We expected to see a dominant role for speech-related brain areas, such as Brocas or Wernickes. However, not even in those areas could we identify an increase in the number of electrodes in the top sets. Furthermore, speech-related processes are usually handled by the left hemisphere of the brain in 90% of right-handed and in ~60–70% of left-handed persons (Chakravarthy, 2018). However, our classification results on subsets of exactly those left-lateralized brain regions were outperformed by our custom-selected electrode positions by the Gray Wolf Optimization algorithm, with differences of up to 20% on average for dataset one. Investigating the different hemispheres and their occurrence in the top set, we observed a minimal dominance of the right hemisphere, which was small enough to be neglected. The handedness of participants in dataset 1 was not available in the notes of the BCI competition. For the two other datasets, all participants were right-handed, which should have led to a dominant presence of electrodes from the left hemisphere.

One possible explanation is that the process of producing imagined speech and the resulting brain activity are widely distributed across the cortex rather than being concentrated in a specific area. The average classification accuracies achieved using the separate subsets of electrodes from the right and left hemispheres support this hypothesis, as they were, on average, identical. This would also clarify the individual results regarding preferences for feature extraction methods and the number of electrodes, which proved to be highly subject-specific.

### 4.4 Impact of dataset selection

Regarding the dataset selection for our evaluation, we outlined the differences between them in the methodology section. We demonstrated that they are as similar as possible, considering that different research groups recorded them. All three studies employed the same headset, electrode layout, recording configuration, and imagined speech paradigm. The setups differed in the language of the words, being Korean for Dataset 1, German for Dataset 2, and English for Dataset 3, which was notable in this case for non-native English speakers. While datasets 1 and 3 used five words for silent repetitions, dataset 2 used 9, which has an impact on the chance level; however, given the reduced number of electrodes, we did not observe a difference in our results. Regarding the imagined speech paradigm, datasets 1 and 2 employed short-block repetitions of the word, which required participants to perform several cued repetitions of the same word in a row, four times for dataset 1 and five times for dataset 2, using a standard training procedure and presenting words on a screen. In contrast, the recording of Dataset 3 involved only single-word repetitions within a specific task, namely, moving a robot through a maze. Taking into account the equal average number of electrodes removed from the three datasets and the same wide variation and distribution of electrodes as shown in the boxplots in Figure 5B, we do not see any evidence for differences between the sets, which would make them not comparable. However, upon closer examination of the average classification accuracies, it remains suspicious that the 5-class dataset recorded with random stimulus presentation (dataset 3) performed worse on average than the 5-class dataset using short-block presentation (dataset 1). The fact that dataset 2, which includes nine classes, was also recorded with short blocks and produced equal results as dataset 3, with only five classes, raises doubts about the short-block stimulus presentation paradigm.

We can once again observe a clear preference for the short-block datasets toward Discrete Wavelet Transformation, while dataset 2, featuring random stimulus presentation, displayed a balanced distribution between feature extraction methods with no evident preference. Our conclusion in this case is that the blocks may induce detectable patterns in the EEG data, leading to label leakage and enhancing the classification process, thereby improving classification results. These effects may be weaker compared to a complete blockwise presentation and probably do not manifest in all participants; however, they are observable and further supported by the broad distribution of results and larger deviations, particularly when employing the wav feature extraction method. However, our findings are preliminary and require further investigation. We can envision a similar setup to Porbadnigk et al. (2009) to shed light on this issue in the future.

### 4.5 Limitations and future studies

The primary contribution and assertion of this work, that imagined speech EEG recordings can be effectively conducted with half the number of electrodes, is not without limitations. As we were unable to identify clear patterns in the reduced sets of electrodes, we cannot conclude which specific positions or areas are best suited for EEG-based imagined speech detection. All our datasets were recorded with 64 electrodes positioned according to the standard 10–20 system, which provides for a widely distributed, homogeneous distribution of the electrodes across the cortex. By systematically reducing the number of electrodes based on classification accuracy and occurrence in top sets, independent of their specific location, we lose the original homogeneous distribution of the 10–20 system and proceed with an individual distribution of electrodes. Our results, therefore, still depend on the layout of a 64-channel setup based on the 10–20 system, albeit with a reduced number of electrodes in this specific layout. Consequently, the results cannot be directly transferred to a 32-channel standard 10–20 layout but must be determined based on an initial measurement with the 64-channel headset as mentioned previously. The overall homogeneous distributions observed in the electrode plots could suggest that a standard 10–20 setup with 32 electrodes might, on average, produce similar results. However, this needs to be confirmed in a direct comparison of 64- and 32-channel measurements of imagined speech for the same subject.

As one of our conclusions is that the analysis should be tailored to the individual, we need to consider that the data evaluated in our experiments has been recorded from individual participants on single days. A closer examination of the individual's data over a specific period could help further investigate the widespread patterns of electrode positions and whether they remain consistent within the data of the same subject over time.

Finally, the primary focus of this study was to reduce the number of electrodes, with classification accuracy as the performance metric. Consequently, the methods were tailored toward electrode reduction rather than source localization. Our analysis followed a systematic stepwise reduction based on classification accuracy, regardless of the overall contribution of each electrode or the specific areas to which it belongs. A more detailed examination of source localization that is independent of channel reduction may, therefore, represent the next step for future studies aimed at reducing electrodes in EEG-based imagined speech BCIs.

## 5 Conclusion

In this study, we systematically investigated electrode reduction for EEG-based imagined speech BCIs across three datasets, each consisting of 15 participants. We evaluated different methods for electrode reduction, identifying Gray Wolf Optimization (GWO) as the most effective. Additionally, our results highlight the strong performance of Extreme Gradient Boosting (XGB) as a classifier for imagined speech EEG data, especially when combined with Discrete Wavelet Transform (DWT) for feature extraction. Building on these insights, we investigated the optimal subsets of electrodes and their spatial distribution across subjects. A fuzzy inference system identified the best-performing electrode sets based on classification accuracy in relation to the number of electrodes. While no single universal set could be determined, which confirms that the optimal number of electrodes is highly subject-specific, we found that in 83% of cases, 30 electrodes could be removed without significantly affecting classification performance. This suggests that commonly used high-density EEG setups with 64+ electrodes are likely oversized and that comparable accuracy can be achieved with approximately 32 electrodes. However, as no distinct electrode positions consistently contributed to classification across subjects, and a homogeneous distribution was observed instead, it remains unclear whether a standard 32-channel EEG following the 10-20 system would produce the same results. Therefore, we recommend beginning with a high-density electrode configuration and optimizing the selection individually. For both research and real-world applications, these findings enable a significant reduction in the number of electrodes by nearly half, improving practicality, reducing setup time, and lowering costs. Future studies should validate these results by comparing high-resolution EEG recordings with individually optimized reduced configurations. Additionally, real-time experiments that dynamically assess electrode contributions could provide a more efficient and adaptive approach to electrode selection.

## Data Availability

Publicly available datasets were analyzed in this study. Dataset one can be found at OSF (https://osf.io/pq7vb/) dataset 2 and Zenodo (https://zenodo.org/records/14645653) dataset 3.

## Footnotes

^1^https://brainvision.com/products/liveamp-64/ Last accessed: 31.01.2025.

^2^https://osf.io/pq7vb/ Last accessed: 31.01.2025.

^3^https://zenodo.org/records/14645653 Last accessed: 31.01.2025.
